# Supplementary material for: Bending Fatigue Behaviour and Fatigue Endurance Limit Prediction of 20Cr2Ni4A Gear Steel after the Ultrasonic Surface Rolling Process
Source: Materials (Basel). 2021 May 12;14(10):2516. doi: 10.3390/ma14102516 (PMC8152014; doi:10.3390/ma14102516)
Supplement: Supplementary file 1 [file materials-14-02516-s001.zip › materials-1157379-supplementary.pdf]

materials

MDPI

Supplementary Materials

Bending Fatigue Behaviour and Fatigue Endurance Limit Prediction of 20Cr2Ni4A Gear Steel after the Ultrasonic Surface Rolling Process

Zhiyuan Wang [1,2], Yangfei Huang [2], Zhiguo Xing [2,*], Haidou Wang [2,3,*], Debin Shan [1], Fengkuan Xie [2] and Jiming Li [2]

[1] School of Materials Science and Engineering, Harbin Institute of Technology, Harbin 150001, China; reincarnational@163.com (Z.W.); shandebin@hit.edu.cn (D.S.)
[2] National Key Lab for Remanufacturing, Army Academy of Armored Forces, Beijing 100072, China; huangyanfei123@126.com (Y.H.); xiefengkuan@126.com (F.X.); lijm0215@163.com (J.L.)
[3] National Engineering Research Center for Remanufacturing, Army Academy of Armored Forces, Beijing 100072, China
* Correspondence: xingzg2011@163.com (Z.X.); wanghaidou@aliyun.com (H.W.)

Table S1. Three-point bending fatigue test data of samples before and after USRP

σ_{max} (MPa)	1656	1422	1326	1230	1073
0	-	77877	385963	776111	2569201
	-	83608	160513	1361390	1596852
	-	96475	202778	637711	2630210
	-	93608	177928	1122622	3000000
	-	65203	450625	856932	1256325
2	89313	114793	401094	3652481	
	87134	257816	778824	2852698	
	55894	190647	689494	1265997	
	65319	107713	303616	2235642	
	74526	230962	419480	3000000	
3	155231	551367	610221	3000000	
	211531	340512	1480729	1569862	
	225541	332007	990242	1120229	
	412689	613224	687198	899520	
	112532	4340521	1526251	2569852	
